# Prevalence, Correlates, and Perception of E-cigarettes among Undergraduate Students of Kathmandu Metropolitan City, Nepal: A Cross-Sectional Study

**DOI:** 10.1155/2023/1330946

**Published:** 2023-11-17

**Authors:** Gayatri Khanal, Abhishek Karna, Suresh Kandel, Hari Krishna Sharma

**Affiliations:** ^1^Department of School of Public Health and Community Medicine, Chitwan Medical College Teaching Hospital (CMC), Bharatpur-13, Chitwan, Nepal; ^2^Centre for Research on Educational, Health and Social Science (CREHSS), Bafal, Kathmandu, Nepal; ^3^College of Medical Science and Teaching Hospital (COMS), Bharatpur-10, Chitwan, Nepal; ^4^National Health Education Information and Communication Centre (NHEICC), Teku, Kathmandu, Nepal

## Abstract

**Introduction:**

The use of e-cigarettes has been increasing globally especially among the youth population due to rigorous advertisement, marketing, and promotion which has become a significant public health concern. Hence, this study is aimed at identifying the prevalence, correlates, and perception of e-cigarettes among undergraduate students of Kathmandu city.

**Methods:**

A cross-sectional study was conducted among 405 undergraduates of capital city of Nepal from April 2022 to December 2022. A two-stage systematic random sampling was used to select the respondents. An anonymous, semistructured, self-administered questionnaire was used for data collection. A descriptive, bivariable, and multivariable analysis was done using SPSS version 20.

**Results:**

The prevalence of ever use and current use of e-cigarettes was 21.2% and 5.9%, respectively. Number of close friends (AOR = 5.23, CI: 1.26, 16.39), number of friends using e-cigarettes (AOR = 7.23, CI: 0.93, 22.82), male sex (AOR = 2.88, CI: 2.15, 10.35), and age (COR = 5.07, CI: 0.93, 8.19) were the major predictors of current e-cigarette usage. Similarly, number of friends using e-cigarettes (AOR = 5.90, CI: 2.15, 10.35), male sex (AOR = 3.53, CI: 2.15, 10.35), age (COR = 4.56, CI: 0.98, 6.24), and place of residence (COR: 5.19, CI: 0.83, 8.02) were the major predictors of ever e-cigarette usage. The prevalence of e-cigarette consumption was higher in males than females (5.4% vs. 0.5%). Approximately, 34.8% respondents had perceived e-cigarettes as a means to help in smoking cessation. 56.5% adults knew that e-cigarettes promoted conventional smoking. Majority (64.7%) of the undergraduate students had presumed e-cigarettes as less harmful to conventional smoking.

**Conclusion:**

Ever use, current use, and misconception on e-cigarettes were widespread among the undergraduate students. Age, male sex, number of close friends, number of peers using e-cigarette, and place of residence were the major predictors for e-cigarette usage. To deal with the increased e-cigarette consumption state, awareness on the harmful addictive properties of e-cigarettes along with its adverse health consequences must be propagated and an appropriate intervention must be implemented.

## 1. Introduction

Due to the rigorous tobacco control initiatives, the global prevalence of smoking tobacco has started to decline significantly in the past decades and the use of new emerging tobacco products like e-cigarettes (EC) has increased dramatically [1] along with the rigorous advertisement, marketing, and promotion through various channels and online platforms [2]. According to a systematic review (2022), the global lifetime and current prevalence of EC were 23% and 11%, respectively [3], whereas in Asia, the lifetime and current prevalence were 16% and 11%, respectively [3]. Cross-sectional studies conducted in various parts of the world have revealed a wide range of EC usage ranging from 1.7% to 15.2% [4–10]. Although the prevalence of e-cigarette consumption in Asia and South East Asia is relatively low compared to other regions, these regions constitute as a vulnerable area for unprecedented growth of e-cigarettes due to little attention and rapidly proliferating market shift [3, 11]. Currently, there are limited data available on prevalence of e-cigarettes in Nepal; however, few studies had also highlighted its burden and uses [12–14].

There has been argument since 2004 on EC appeared on the market that they are less harmful to conventional cigarettes and are taken as alternatives to quit traditional smoking, which further led to increased usage [2, 15]. Moreover, EC markets attract younger people easily because EC are produced with a variety of flavors, such as mint, candy, fruit, or chocolate, and in a variety of forms, often resembling pens, tech gadgets, and other everyday items [2, 16]. Center for Disease Control (CDC) and World Health Organization (WHO) have declared that most e-cigarettes were filled with nicotine, which is addictive and toxic. Nicotine exposures are more harmful to adolescents and young adults because they can alter brain development, increase risk of cardiovascular diseases, and damage the lungs as well [17]. Similarly, growing literature had showed that gender, age, school location, academic performance, peer and parental influence, number of friends using e-cigarettes, and knowledge and perception of EC have significant linkage with the use of EC [4, 6, 9, 10, 18, 19].

The EC industry is emerging in Nepal and is run by three major authorized dealers: Vape City, Guff and Puff, and Vapemandu [12–14]. The major customers are mainly adolescents and youths of Kathmandu valley. Moreover, the streets of Kathmandu are lined up with several retailers, parlours, and online shops which offer EC in a convenient way [12–14], thus becoming an emerging public health issue in Nepal [20]. Despite the implementation of tobacco product control and regulatory directives, Nepal has imported more than 18 billion electronic cigarettes and other similar vaporizing devices accounting to more than Rs 11 million in fiscal year 2021/2022 [12]. The existing weakness in tobacco regulatory programs of Nepal has allowed the EC business to inflate rapidly with emergence of new hybrid products especially in the big cities like Kathmandu [12, 20].

E-cigarette usage and health of adolescent and youth have evolved as a potential threat in Nepal thereby directing and promoting more medical research in this field. In spite of this, limited researches have been conducted in Nepal. Therefore, this novel study was performed to estimate the prevalence of EC usage and its associated factors in Nepal. This study will generate evidence and fulfill the knowledge gap as well as highlight the new challenges of tobacco consumption to the policy makers. Hence, this study would prove to be worthy enough not only in assessing the prevalence and covariates of EC usage but also in investigating the students' perception on EC usage.

## 2. Methodology

### 2.1. Study Design, Area, and Participants

E-cigarette business is proliferating in bigger cities of Nepal, especially in the capital city (Kathmandu) of Nepal [12, 13]. A cross-sectional study was operated in Kathmandu valley from April 2022 to December 2022 including 270 undergraduate students [21].

### 2.2. Selection of the Participants

The population proportion formula *N* = *Zpq*/*L*^2^ was used to calculate sample size with the assumption of 50% population proportion using e-cigarettes within 95% confidence interval at 5% allowable error with critical value of *Z* = 1.96 [22]. The calculated final sample size was 405 after adding the 5% nonresponse rate. Two-stage systematic random sampling technique was used for selection of the participants. In the first stage, list of undergraduate colleges was retrieved from educational section of Kathmandu metropolitan office [21]. There were 270 colleges, out of which nine undergraduate colleges were selected by using systematic random sampling method. To perform systematic sampling, we calculated the *k*th interval (270/9 = 30^th^) and the first college was selected by lottery method from 1 to 30 colleges, and then, other colleges were selected at 30^th^ interval. In the second stage, list of undergraduate students in each of the selected colleges was retrieved. Out of the 2495 enrolled students, 405 were selected at every 6^th^ student interval. First participant was selected in a similar way as mentioned above in the 1^st^ stage. Students who were absent on the day of data collection were excluded from the study, and the next nearest was chosen as a respondent. Only those respondents who were currently pursuing undergraduate degree in colleges of Kathmandu city and willing to give verbal informed consent were considered as respondents.

### 2.3. Data Collection Technique

An anonymous, semistructured, self-administered questionnaire was developed after the literature review and support of various other similar studies [4–8, 18, 23, 24]. Both English and Nepali versions of the questionnaires were prepared and used according to the familiarity of students. The questionnaire was pretested among 10% of the total sample size in two undergraduate colleges of Kathmandu city. Necessary modifications and improvisation of the questionnaire were done after the feedback from the pilot test. Researchers themselves were involved in collecting data. Respondents were approached after taking approval from the college authority. Before the data collection, research participants were clearly informed about the type of research, objectives, and its benefits in the near future.

### 2.4. Study Variables

#### 2.4.1. Dependent/Outcome

In this study, ever use of EC is defined as the use of EC in any point of life, and current use of EC as any use in the previous 30 days. Current use of electronic cigarettes is represented by a question “During past 30 days, how many days did you use electronic cigarette?”; if the answer is 1 day or more, then it is considered as “current user”; if the answer is 0 day, then it is considered as “noncurrent users.” Similarly, ever use of e-cigarettes was estimated by a question “Have you ever tried e-cigarettes even one or two puffs?” and the answer was coded as 1 “yes” or 2 “no.”

#### 2.4.2. Independent/Predictive

The age was categorized as 18-19 years and 20-26 years. Sex of the respondent was categorized as male or female. Religion was divided into Hindu and non-Hindu. Ethnicity of the respondents was coded as Brahmin/Chhetri and second category for others. Based on the information of marital status, respondents were categorized as together or separated/divorced/deceased. Educational status of the father and mother was coded as illiterate and literate. Types of family were allocated as nuclear or joint. The family income of the respondents was divided into two categories: “below 50,000 Rs per annum” and “above 50,000 Rs per annum.” Residential place was considered as city or village. Having older siblings was considered as “yes” or “no,” and number of close friends was coded as “3 or less than 3” and “more than 3.” Age at using e-cigarette use was coded as “<18 years age” and “>18 years age.” Number of friends who use e-cigarettes was categorized as zero, 1, and more than one. Current smoking habit of family members, current e-cigarette use of family members, current smoking habit of older siblings, and current e-cigarette use of older siblings were categorized into “yes” and “no.” Perception on EC was measured by independent five (yes, no) statements. These include the following: (1) EC help in smoking cessation, (2) EC promotes conventional smoking, (3) EC should be banned, (4) EC should be regulated like other tobacco products, and (5) EC are harmful to our health.

### 2.5. Data Processing and Analysis

Data entry was done using EpiData version 4.6. Data analysis was done using IBM Statistical Package for the Social Sciences (SPSS) version 20. The descriptive findings were mean, standard deviation, percentages, and frequency. To determine the independent factors associated with current and ever EC use, significant variables (*p* ≤ 0.05) of chi-square test were included for bivariable logistic regression analysis. Those variables having significant association (*p* ≤ 0.05) in bivariable regression analysis were included for multivariable logistic regression analysis. The Nagelkerke R Square and variation inflation factor (VIF) tests were performed to determine the goodness of fit for multivariate logistic regression. There was no multicollinearity among independent variables.

### 2.6. Ethical Approval and Consent to Participate

Ethical approval of this study was granted from Institutional Review Board of Chitwan Medical College (ref. no. CMC-IRC/078/079-231). Concerned stakeholders were officially contacted with letters, and permission was obtained from all levels. The study was clearly explained to participants. Verbal informed consent was obtained from the undergraduate students.

## 3. Results

The mean age of the respondents was 20.46 ± 2.008 years. 57.8% participants were female. Most of the participants (85.7%) were Hindu. Ethnically, nearly half (52.6%) of the respondents were Brahmin or Chhetri. 12.3% and 29.6% of the respondents' father and mother were illiterate, respectively. Approximately, two-thirds (67.9%) of the respondents had older siblings, and 58.5% respondents had more than three close friends. More than three-fourths (79.8%) of the respondents were staying in urban areas. 8% of the respondent parents were separated/divorced/deceased (Table 1).

The overall prevalence of ever use EC was 21.2%. The prevalence of ever use EC was higher among boys as compared to girls (17.5% vs. 3.7%). Similarly, the prevalence of current use EC was 5.9% (boys, 5.4%, and girls, 0.5%). More than half (54.8%) of the respondents had heard about e-cigarettes.

More than one-third (34.8%) of the respondents presumed that e-cigarettes help in smoking cessation. More than half (56.5%) of the participants were unaware of the fact that e-cigarettes promoted conventional smoking. Nearly one-third (33.6%) of EC users had belief that it should not be banned. Nearly, three-fifths (57.5%) of the respondents stick to the opinion that e-cigarettes should not be regulated like other tobacco products. Out of the 89.1% EC users, who admitted that EC were harmful, only one-fourth (24%) knew that it was less harmful as compared to conventional smoking.

The major causes for ever use of e-cigarettes were persuasive suggestions by close friends (80.6%) and sense of better taste (50%) compared to conventional smoking. The reasons behind consuming e-cigarettes were variety of flavors (75%), better taste (62.5%), perception of not or less harmful to health (58.3%), stylish and fancy looking (54.2%), assumption of ease to quit smoking (54.2%), and convenience to use anywhere because of no smoke (41.7%). Less than one-third (27.9%) of the undergraduates who had ever tried EC turned up being current users. Majority (74.4%) of the e-cigarette users had purchased it from the valley shops followed by online shops (24.4%). 27.9% of the undergraduate who tried EC was shifted to current EC smokers (Table 2).

Table 3 presents the multiple regression analysis of current use of e-cigarette and ever use of e-cigarette with COR and AOR values. Regarding current use of EC, undergraduates of age 20 to 26 years (COR = 5.07, CI: 0.93, 8.19), male sex (COR = 6.48, CI: 1.82, 12.19; AOR = 2.88, CI: 1.34, 10.67), undergraduate students with less or equal to three close friends (COR = 3.33, CI: 1.76, 11.03; AOR = 5.23, CI: 1.26, 16.39), and undergraduate with two or more than two friends currently on EC (COR = 4.23, CI: 1.73, 14.45; AOR = 7.23, CI: 0.93, 22.82) were 7.23 times more likely to vape e-cigarettes. Similarly, regarding ever use of EC, undergraduates of age 20 to 26 years (COR = 4.56, CI: 0.98, 6.24), male undergraduates (COR = 6.82, CI: 3.56, 13.08; AOR = 3.53, CI: 2.15, 10.35), urban resident (COR = 5.19, CI: 0.832, 8.028), and undergraduates with two or more than two friends currently on EC (COR = 15.56, CI: 8.05, 30.09; AOR = 5.90, CI: 1.90, 14.67) were more likely to ever use EC (Table 3).

## 4. Discussion

In this study, interdependence of prevalence of EC uses and general characteristics of the undergraduate students who uses EC was assessed. Above analysis displays positive association between use of EC and number of close friends, number of friends using EC, male sex, age, and place of residence. Overall prevalence of ever use and current use of EC was 21.2% and 5.9%, respectively. The most common reasons reported for EC use were suggestions by a close friend, flavor variety for better taste, and perceiving EC as not or less harmful to health.

The prevalence of ever use EC (21.2%) in this study was comparable to the study conducted by Qi et al. in 2022 (14.9%) [6], and Aljandaleh et al. in 2020 (26.9%) [7]. Studies done by Deng et al. in 2022 [5], Patanavanich et al. in 2021 [4], and Jeon et al. in 2016 [18] reported a lower proportion in EC usage of 4.5%, 7.2%, and 12.6%, respectively. In contrast to this study, the study done by Peckham et al. in 2020 (70.3%) [24] and Wamamili et al. in 2020 (40.5%) [8] revealed a higher burden of EC usage. Similarly, the prevalence of current usage of EC (5.9%) in our study was similar to the reports shared in studies by Patanavanich et al. in 2021 (5.9%) [4], Wamamili et al. in 2020 (6.1%) [8], and Tavolacci et al. in 2016 (5.7%) [9]. In contrast to this study, a higher burden of current EC consumption was observed in various other studies by Aljandaleh et al. in 2020 (15.2%) [7] and Kurdi et al. in 2021 (14%) [10] while a lower proportion of users was noticed in studies conducted by Deng et al. in 2022 (1.7%) [5] and Qi et al. in 2022 (3%) [6]. The dissimilarity in the ever use and current use of e-cigarettes might be due to variation in survey time, location, marketing norms/strategies, availability/affordability, and awareness status. Moreover, the varied definitions of ever and current use of e-cigarettes for measuring the prevalence of EC found in different studies may further lead to contrasting results on ever and current uses of EC. Both terms (ever use and current use) are frequently used interchangeably even though there is clear demarcation between them. The chance of shifting permanently to current users is much higher among those who have been exposed EC once in their lifetime compared to those who have never been exposed to EC [25].

Current study had supported that students who were adults rather than younger were found more likely to consume EC. This finding was contradicted by Etim et al.; the study conducted in 2020 stating that the probability of e-cigarette usage gradually increases over the age of 15 years and then decreases by 18 years [26]. Possibilities of varied results could be due to the differences in study area (Kathmandu vs. California), parental control/guidance over their children, and social-cultural norms and values. Moreover, in Nepal, child freedom is decisive to the parents until they are married or separated. Current study showed that those having a small close friend circle were more likely to end up using EC, which was not the case seen in several other studies [4, 18, 27]. The major reason behind it could be related to sociocultural norms and context of Nepal where parents usually follow strong restriction practices on tobacco-related product usage. Hence, forming a small confined circle will definitely ensure more privacy for smoking. However, regarding the number of friends using EC, the scenario was different. Those with a higher number of friends (2 or more) were more likely to use EC. Number of close peers using EC was associated with higher odds of current e-cigarette usage. These findings were similarly reported in various other studies by Patanavanich et al. in 2021 [4], Jeon et al. in 2016 (21.6%) [18], Kurdi et al. in 2021 (14%) [10], and Choi and Forster [27] and Etim et al. in 2020 [26]. Furthermore, use of EC among the boys was significantly higher as compared to girls which was similarly recorded in various studies conducted in different parts of the world [4, 6, 8, 10]. For a simpler reason, male youths are easily attracted by its flavor and smokeless character [19, 28, 29]. Moreover, boys have a more risk-taking behavior than girls and therefore end up in trying a new electronic gadget like EC. In our study, urban residents had more chances of using EC, which was not evident with Lewis-Thames et al.'s study in 2020 stating that there is no difference for experimenting EC among urban-rural residents [30].

The predominant reasons for EC use in the present study were persuasive suggestions by a close friend, suitable for flavor variety/better taste, false assumption of innocuous to health, easiness in reducing or quitting cigarettes, and convenient anywhere for smoke building smoke tricks. The top reasons for initiation of e-cigarette for the lifetime were misguidance from friends, curiosity of different flavors and taste, and intentional untroubled quitting of cigarettes [19, 24, 28, 29]. E-cigarettes are an innovative product, introduced to the Nepalese market from 2014 onwards [9]. Moreover, they are broadly advertised and covered in the popular media [12–14], disseminating false information in such a way that EC looks lucrative and attractive [31].

The current study revealed that more than half (56.5%) of the undergraduate students disagree with the fact that e-cigarettes promote or increase conventional smoking. However, a report published by WHO had declared that e-cigarette used by adolescents and youth can double their risk of smoking cigarettes [25]. Similarly, ample evidence has shown that EC use was strongly associated with the consumption of other tobacco products among the youth and adolescents, which have become a public health concern [17, 25, 32]. Undergraduate students perceived e-cigarettes as an alternative means of reducing or quitting conventional smoking. Our findings were consistent with earlier studies shared by Patanavanich et al. in 2021 [4], Wamamili et al. in 2020 [8], Baltz et al. in 2019 [19], and Choi and Forster in 2013 [27]. This perception among the youth's had led to an increase in the consumption of EC [33]. Majority (64.7%) of undergraduate students had perceived EC as less harmful (64.7%) than conventional smoking. The above finding was similarly mentioned in line with various other studies conducted by Qi et al. in 2022 [6], Wamamili et al. in 2020 [8], Baltz et al. in 2019 [19], Choi and Forster in 2013 [27], and Jiang et al. in 2019 [34]. WHO report on the global tobacco epidemic (2021) addressed that e-cigarettes were promoted aggressively as safer and smoke-free alternatives to conventional cigarettes, which easily penetrates the youth's mind [25]. A study conducted in the Unites States had found that the innocuous perception of e-cigarettes among the young adults had accelerated the e-cigarette usage [35]. This evidence is an eye-opening concern for the undergraduates in shaping their behavior towards the use of EC. The most common reasons behind misperceptions were smokeless characteristics of EC and lucrative marketing strategies focusing mainly on the benefits rather than side effects which could contribute to global burden for tobacco control [2, 15, 16, 25]. It should be emphasized that cigarette smoking as well as EC use is a learned behavior influenced by various components, which passes through various stages of preparation, initiation, experimentation, regular and long-term use, and addiction [36]. This process of acquisition is largely influenced by EC industry advertisement and marketing techniques for getting promoted as a safer alternative to conventional smoking. This study revealed that more than half (54.8%) of the participants were aware of EC. The prevalence of e-cigarette awareness in this study (54.8%) was lower than that reported by Deng et al. (94.2%) study in 2022 [5], but higher than Jeon et al. in 2016 (21.6%) [18] and Regan et al.'s study in 2010 (32.2%) [23]. The variability in awareness on e-cigarettes is may be attributed to city-specific market factors, varying tobacco control policies and regulations, differences in survey timing, and directions of EC awareness and trial/use in each specific setting. One-fourth (25%) of the current EC users also had the habit of consuming conventional smoking, which was also supported by Goniewicz et al.'s study in 2014 (21.8%) [37]. Jeon et al.'s study in 2016 [18] reported a fourfold increase (96.3%) in dual use of cigarettes, while other studies by Lee et al. in 2014 [38], Tavolacci et al. in 2016 [9], and Kim et al. in 2020 [32] reported a lower proportion of dual usage with 8%, 14.5%, and 10.21%, respectively.

The current analysis has several strengths. This study has been conducted in a capital city of Nepal where the potential of EC use is high. This study is a pioneer study in the Nepalese context regarding perception and burden of EC usage. The present study has limitations, as the study was conducted in the capital city (Kathmandu) using random sampling technique which may not represent the scenario as a whole. This is a cross-sectional study requiring the adjusted ratio. Hence, the data generated from this study constrains make causal inferences about the relationship found in the research. Participants own responses to e-cigarette use may be biased or might be underreported, as this process was based on self-reporting. More researches need to be conducted in order to identify the causal relationship trends of EC use in a large population. Despite the limitations, the current study provides a valuable insight into the prevalence, perception, and correlates of EC use among the undergraduate from Kathmandu, Nepal.

## 5. Conclusions

Despite e-cigarette ban in Nepal, there was a high burden of ever use and current use of EC among the undergraduate students. Participants had ample misconceptions and false belief regarding the EC usage. Age, sex, number of close friends, number of peers using EC, and awareness on EC were the major predictors for EC consumption among undergraduate students. These findings suggest that strategic interventions are needed to tackle the increased burden of e-cigarette use and appropriate awareness on its addictive properties along with adverse health consequences. Furthermore, more detailed research evidence need to be generated on the large scale is crucial.

## Figures and Tables

**Table 1:** Distribution of background-related characteristics of study population.

Variables	Frequency (*n* = 405)	Percentage (%)
Age		
18-19	155	38.3
20-26 Mean age ± SD: 20.46 ± 2.008, range 18–26	250	61.7
Sex		
Male	171	42.2
Female	234	57.8
Religion		
Hindu	347	85.7
Non-Hindu	58	14.3
Ethnicity		
Brahmin/Chhetri	213	52.6
Others	192	47.4
Type of family		
Nuclear	271	66.9
Joint	134	33.1
Family income (Rs)		
Below 50,000	257	63.5
Above 50,000	148	36.5
Marital status of parents		
Together	373	92
Separated/divorced/deceased	32	8
Educational status of father		
Illiterate	50	12.3
Literate	355	87.7
Educational status of mother		
Illiterate	120	29.6
Literate	285	70.4
Place of residence		
City	323	79.8
Village	82	20.2
Older siblings		
Yes	275	67.9
No	130	32.1
Number of close friends		
3 or less than 3	168	41.5
More than 3	2377	58.5

**Table 2:** Description of e-cigarette-related factors and perceptions including all participants.

Statements and responses	Frequency (*n* = 405)	Percentage (%)
Ever use of EC	86	21.2
Boys	71	17.5
Girls	15	3.7
Current use of EC	24	5.9
Boys	22	5.4
Girls	2	0.5
Heard about EC	222	54.8
Current smoking habit of family members	107	26.4
Current EC use of family members	19	4.7
Current smoking habit of older siblings	33	8.1
Current EC use of older siblings	23	5.1
*Perception on EC*		
EC help in smoking cessation	141	34.8
EC promotes conventional smoking	176	43.5
EC should be banned	269	66.4
EC should be regulated like other tobacco products	172	42.5
EC are harmful for our health	361	89.1
*If yes, degree of harmfulness of e-cigarettes is as compared to conventional smoking*		
More harmful	143	35.3
Equally harmful	165	40.7
Less harmful	97	24
*No. of friends using EC*		
Zero	324	80
One	27	6.8
More than 1	33	8.2
*Reasons for current use of EC # multiple response*	Frequency (*n* = 24)	%
Variety of flavors	18	75
Better taste	15	62.5
Not or less harmful for health	14	58.3
Become stylish and fancy	13	54.2
Easier to cut down or quit smoking	13	54.2
Can use anywhere because of no smoke	10	41.7
Current use of EC along with conventional cigarette	6	24
Use of conventional smoking prior to EC	10	41.7
*Reasons for ever use of EC # multiple response*	Frequency (*n* = 86)	%
Suggestions by close friend	50	80.6
Heard of better taste	43	50
Makes stylish and fancy	33	38.4
Shift from conventional smoking	15	24.2
To quit conventional smoking	27	31.4
*Where do you purchase e-cigarettes? # multiple response*		
Online shops	21	24.4
Ask close ones to bring from foreign country	18	20.9
Valley shops	64	74.4
Students who tried e-cigarettes shifted to current users	24	27.9

**Table 3:** Odds ratio and 95% CI for various factors associated with current use and ever use of e-cigarettes.

Dependent: current use of EC		^a^OR (bivariable)	*p* value	^b^OR (multivariable)	*p* value
Age	18-19	1		1	
	20-26	5.07 (0.93, 8.19)	0.036^∗^	2.03 (0.48, 7.89)	0.51

Sex	Male	6.48 (1.82, 12.19)	0.002^∗^	2.88 (1.34, 10.67)	0.008^∗^
	Female	1		1	

No. of friends using e-cigarettes	0 or 1	1			
	≥2	4.23 (1.73, 14.45)	≤0.001^∗^	7.23 (0.93, 22.82)	≤0.001^∗^

Number of close friends	≤3	3.33 (1.76, 11.03)	0.020^∗^	5.23 (1.26, 16.39)	0.010^∗^
	>3	1		1	

Dependent: ever use of EC					
Age	18-19	1			
	20-26	4.56 (0.98, 6.24)	0.050^∗^	1.6 (2.24, 14.11)	0.74

Sex	Male	6.82 (3.56, 13.08)	≤0.001^∗^	3.53 (2.15, 10.35)	≤0.001^∗^
	Female	1		1	

No. of friends using e-cigarettes	0 or 1	1		1	
	≥2	15.56 (8.05, 30.09)	≤0.001^∗^	5.90 (1.90, 14.67)	≤0.001^∗^

Place of residence	Urban	5.19 (0.83, 8.02)	0.040^∗^	2.46 (0.56, 7.56)	0.58
	Rural	1			

^∗^Statistically significant at *p* < 0.05. 1 represents reference category. ^a^Crude odds ratio. ^b^Adjusted odds ratio. Note: current use of EC was adjusted with age, number of close friend, and number of friends using e-cigarettes, and ever use of EC was adjusted with sex, number of friends using e-cigarettes, place of residence, and age.

## Data Availability

All the data are available upon reasonable request from the corresponding author.

## Abbreviations

WHO: World Health Organization
CDC: Center for Disease Control
VIF: Variation inflation factor
SPSS: Statistical Package for the Social Sciences
EC: E-cigarettes
AoR: Adjusted odds ratio
CoR: Crude odds ratio.
